# Comparative Efficacy of Antifungal Agents Used in the Treatment of Oropharyngeal Candidiasis among HIV-Infected Adults: A Systematic Review and Network Meta-Analysis

**DOI:** 10.3390/jof7080637

**Published:** 2021-08-05

**Authors:** Shamala Gopal Rajadurai, Mari Kannan Maharajan, Sajesh K. Veettil, Divya Gopinath

**Affiliations:** 1 School of Postgraduate Studies, International Medical University, Kuala Lumpur 57000, Malaysia; srsham17@gmail.com; 2 Department of Pharmacy Practice, School of Pharmacy, International Medical University, Kuala Lumpur 57000, Malaysia; marikannan@imu.edu.my; 3 Department of Pharmacotherapy, College of Pharmacy, University of Utah, Salt Lake City, UT 84112, USA; Sajesh.Veettil@pharm.utah.edu; 4 Clinical Oral Health Sciences, International Medical University, Kuala Lumpur 57000, Malaysia

**Keywords:** oropharyngeal candidiasis, oral candidiasis, HIV, antifungal agents, prevention, treatment, systematic review, network meta-analysis

## Abstract

The objective of this study was to assess the comparative efficacy and safety of different antifungal agents used for the treatment of oropharyngeal candidiasis (OPC) in adult patients with HIV. A systematic search was performed on the four major databases (Medline, Embase, CENTRAL and Scopus) to identify randomized controlled trials (RCTs) that evaluated the efficacy of antifungal agents in HIV patients with OPC. A network meta-analysis was performed from the data extracted from the selected studies. The agents were ranked according using surface under the cumulative ranking (SUCRA). The Grading of Recommendations, Assessment, Development and Evaluation (GRADE) approach was used to determine the quality of evidence. A total of 15 trials were included in the quantitative analysis involving the data from a total of 2883 participants. Fluconazole was ranked as the most effective antifungal agent to achieve clinical cure (SUCRA = 0.87) in OPC followed by posaconazole and itraconazole. Posaconazole was ranked the most efficacious agent in achieving mycological cure (SUCRA = 0.81), followed by fluconazole. While nystatin was ranked the safest, the effect estimates of none of the other systemic antifungal agents were significantly higher than fluconazole. Based on the available evidence, fluconazole can be considered as the most effective drug in the treatment of OPC among HIV-infected adults and has a favorable safety profile, followed by posaconazole.

## 1. Introduction

HIV-infected patients often battle opportunistic infections due to the nature of the disease, which impairs their immune system, and oropharyngeal candidiasis (OPC) happens to be one of those infections [1,2]. The beginning of the ‘highly active antiretroviral therapy’ (HAART) era in 1996 saw a drastic decrease in the incidence of OPC and other opportunistic infections, and this was correlated with the fact that HAART led to an increase in the CD4^+^ count [3]. After the introduction of HAART, of the incidence of OPC was only observed among patients, who had not responded to the treatment effectively [3], hence making it a crucial predictor of disease progression [4]. However, OPC is the most common oral opportunistic infection, and is still one of the health concerns of HIV patients [5,6].

OPC can have a negative impact on the quality of life (QoL) [7], as it causes dysgeusia, burning sensations in the mouth and discomfort in the oral cavity [8]. This negative impact on HIV patients makes the treatment more challenging as the patient may present with difficulty in swallowing oral medication, which could potentially affect their compliance with HAART or any other medications [9]. Without an appropriate treatment, OPC can progress to esophageal candidiasis, and this would potentially reduce the intake of food and nutrients, which would further deteriorate the health condition of HIV patients [10,11,12]. Therefore, the rapid diagnosis and effective management of OPC in HIV-infected patients is essential.

There is a variety of antifungal agents available for the treatment of OPC in the HIV-infected adult population, including systemic and topical formulations. Most of the available RCTs reported a pairwise comparison. Based on the available reports, clotrimazole is the most commonly used antifungal agent for the management of OPC [10,11,12,13]. Koletar et al. reported fluconazole to be significantly more effective than clotrimazole in the treatment of OPC [13]. Redding et al. reported similar findings; however, the difference in effectiveness was not statistically significant [14]. The effectiveness of itraconazole was found to be as effective as the fluconazole treatment regime [15]. Comparing itraconazole to ketoconazole, itraconazole was found to be more efficacious than ketoconazole; however, this difference was not statistically significant [16]. However, when comparing fluconazole against ketoconazole, fluconazole was found to be superior [17,18,19]. Posaconazole was reported to be as efficacious as fluconazole, although it is worth taking note that with posaconazole, more patients remained symptom-free and fewer patients had clinical relapse compared to fluconazole [20]. Based on the published reports, not all antifungal agents were compared to each other directly in any of the RCTs. Considering the effectiveness and safety of antifungal agents to treat OPC among HIV-infected patients, there is a lack of comprehensive evidence. Hence, choosing the most effective intervention for the management of the OPC in HIV-infected adults is a great challenge in clinical practice.

Unlike conventional meta-analysis, network meta-analysis would allow a direct and indirect comparison of different treatments, which would enable the production of more accurate detail on which antifungal is the most efficacious and safest [21]. Such information would help clinicians and supportive health care providers in decision making when there are limited resources or access to antifungal agents. Thus, the objective of this study was to compare the efficacy and the safety profile of the different antifungal agents used to treat OPC in the adult HIV population as well as to rank them accordingly.

## 2. Material and Methods

This study was designed as a systematic review and network meta-analysis (NMA) following guidelines from the Cochrane Handbook for Systematic Reviews of Interventions [22]. The findings of this study were reported as recommended by the Preferred Reporting Items for Systematic Reviews and Meta-Analyses (PRISMA) extension statement of NMA [23]. This study was registered on PROSPERO with the registration number CRD42020202356.

### 2.1. Search Strategy and Study Selection

Eligible studies for this review were identified by first developing a search algorithm in Medline. Once this was developed, it was then modified and applied to the other three databases, i.e., Embase, Cochrane Central Register of Controlled Trials (CENTRAL) and Scopus. All studies published up to 15 October 2020 were included in the preliminary search. The detailed search strategy is provided in the supplementary material (Supplementary Tables S1 and S2).

### 2.2. Inclusion Criteria

The studies included were randomized controlled trials (RCTs), which met the following inclusion criteria:

1. Participants had to be HIV-infected adults with OPC;
2. Interventions were any class of antifungal agents (at any dose), or complementary medicine tested for the treatment of OPC;
3. Comparisons were other classes of the antifungal agent or other active interventions which were used;
4. The primary outcome was the number of patients who achieved clinical cure;
5. The secondary outcomes were the number of patients who achieved mycological cure from OPC, adverse events and the rate of OPC relapse.

Clinical cure is defined as the resolution of signs and symptoms of OPC, whereas mycological cure is defined as the eradication of candida from the patient’s oral cavity upon completion of the respective treatment.

### 2.3. Data Extraction and Quality Assessment

Data extraction was carried out independently by two reviewers (S.G.R., D.G.). The reviewers resolved disagreements by discussion. Information from each study was extracted from each of the eligible studies and separated into the following sections: study characteristics, population characteristics, intervention characteristics and outcomes. For all outcomes, the initial number of participants randomized to each trial arm was used to perform the analysis, irrespective of how the authors of the original trials had analyzed the data (intention-to-treat principle) [23].

The risk of bias (RoB) was assessed for each study using the updated version of the Cochrane risk-of-bias tool for randomized trials (RoB 2) [24] and any disagreement which arose was settled by a discussion between the reviewers, and if not, resolved independently by a third reviewer. 

### 2.4. Data Synthesis and Statistical Analysis

For direct comparisons, we performed a standard pairwise meta-analysis using a DerSimonian and Laird random effects model to estimate the pooled relative risk (RR) and 95% confidence intervals (CIs), summarizing the efficacy of each active and control intervention tested [25]. A random effects network meta-analysis (frequentist approach) using a consistency model was applied to generate the available evidence by combining direct and indirect evidence from different studies [26,27]. If a direct comparison was based on two or more studies, we assessed the heterogeneity between trials using the I^2^ statistics; an I^2^ estimate ≥50% was interpreted as evidence of substantial levels of heterogeneity [28,29]. Network inconsistency was evaluated using a global inconsistency test by fitting design-by-treatment in the inconsistency model [30,31]. We used the surface under the cumulative ranking curve (SUCRA), which estimates the probabilities for all treatments to obtain a treatment hierarchy. We reported the relative ranking of interventions on efficacy and safety outcomes as their SUCRA, ranging from 1, indicating that the treatment has a high likelihood of being the best, to 0, which indicates the treatment has a high likelihood of being worst [32]. Publication bias was examined with a comparison-adjusted funnel plot [33]. Stata version 15.0 (Stata Corp, College Station, TX, USA) was used for statistical analysis and graph generation.

Additionally, the Grading of Recommendations, Assessment, Development and Evaluation (GRADE) approach was used to rate the quality of evidence (high, moderate, low and very low) of estimates derived from NMA [34].

## 3. Results

### 3.1. Search Results

**Figure 1:**
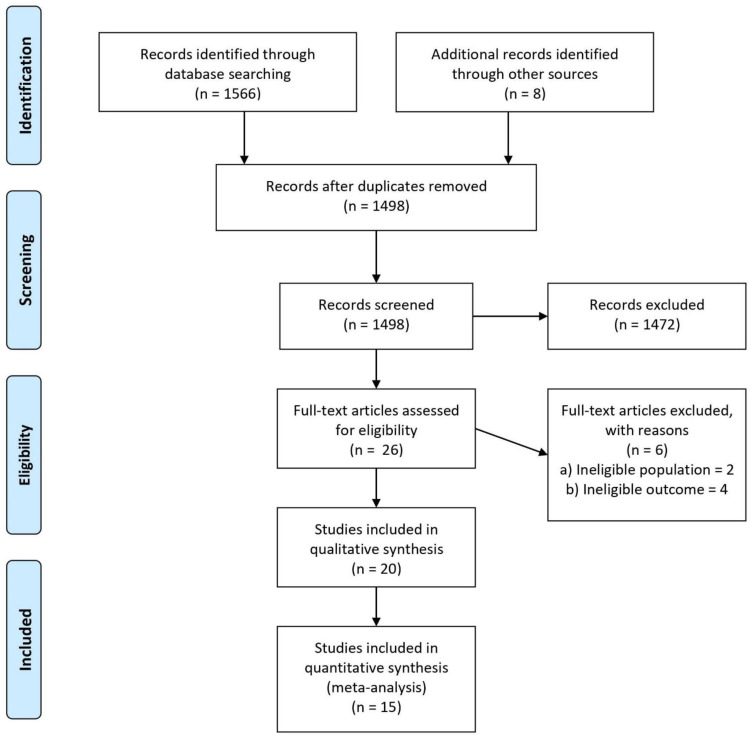
PRISMA flow diagram for selection of studies.

The search for randomized controlled trials was conducted in the four databases and it resulted in the identification of 1566 articles (Supplementary Tables S1 and S2), of which 76 duplicate articles were removed using a reference manager, leaving us with 1490 articles from the preliminary search. An additional 8 articles were identified from relevant review articles and added to the list, totaling to 1498 articles for the title and abstract screen. After the title and abstract screen, we selected 26 articles for full text screening and eligibility. Upon the retrieval of the full text, we then screened them once more, from which an additional six articles were excluded; two were excluded on the grounds of the ineligible study population [35,36] as the results reported were from a mixed population of patients with OPC and EC, while the remaining four were excluded [18,37,38,39] as they reported ineligible outcomes, which did not meet our desired outcome (Supplementary Table S3). Fifteen out of the twenty studies were included for quantitative analysis. The PRISMA flow diagram is as shown in Figure 1. The study characteristics and outcomes of these trials are depicted in Table 1.

### 3.2. Study Characteristics

A total of 20 studies were included in the qualitative analysis, and 15 for quantitative analysis, involving 2883 participants all together (Figure 1). Three out of the twenty studies compared the effectiveness and safety of fluconazole capsules at the daily dose of 100 mg with clotrimazole troches at the dose of 10 mg five times a day [13,14,40] for 14 days of treatment. In one study, all the participants were male [14], while for the other two studies [13,40], the majority of them were male participants. Another three trials compared the safety and efficacy of fluconazole with itraconazole in treating OPC. One of these studies compared a fluconazole capsule given at a single dose of 150 mg with oral itraconazole given at a daily dose of 100 mg for 7 days [41], while the other two studies compared two different itraconazole dosing regimens (7 days vs. 14 days and daily dosing against twice-a-day dosing) with the standard 14-day fluconazole regimen [15,42]. Most participants in all three studies were male. The following drug comparisons had two studies with similar drug pairings: itraconazole compared with ketoconazole [16,43], clotrimazole troche compared with itraconazole [44,45], and single-dose fluconazole compared with a daily dosing regimen of fluconazole [46,47]. Among the remaining six studies, each study compared the following drug regimens: two different doses of a discontinued antifungal agent (D0870) [48]; miconazole buccal tablet with clotrimazole troche [49]; fluconazole and nystatin [50]; alcohol-based melaleuca with alcohol-free melaleuca [51]; miconazole buccal tablet with ketoconazole [52]; posaconazole with fluconazole [20]; gentian violet with nystatin oral suspension [53]; and a comparison of effectiveness in treating OPC with lemon juice/lemongrass and gentian violet [54]. Two out of the twenty studies had three-arm comparisons [15,42] while the rest were two-arm comparison studies.

### 3.3. Risk of Bias

**Figure 2:**
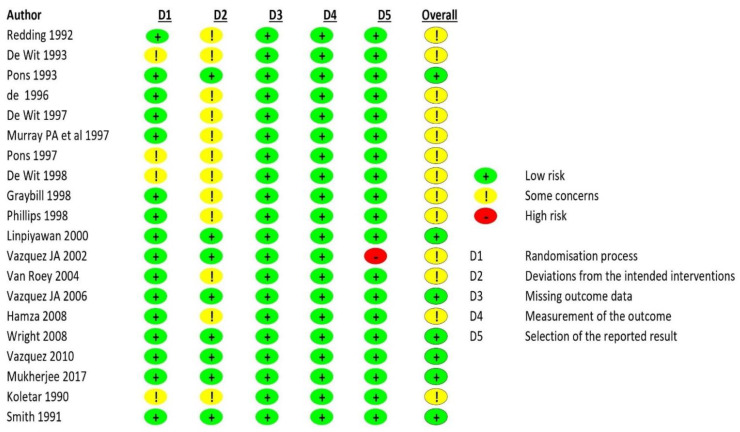
Risk of bias.

Seven [20,40,43,45,49,53,54] out of twenty studies that were included were at low risk of bias, while the remaining thirteen had some concerns regarding the risk of bias. The summary of this assessment is shown in Figure 2.

### 3.4. Efficacy of Antifungal Agents Used in the Treatment of OPC to Achieve Clinical Cure (Network Meta-Analysis)

**Figure 3:**
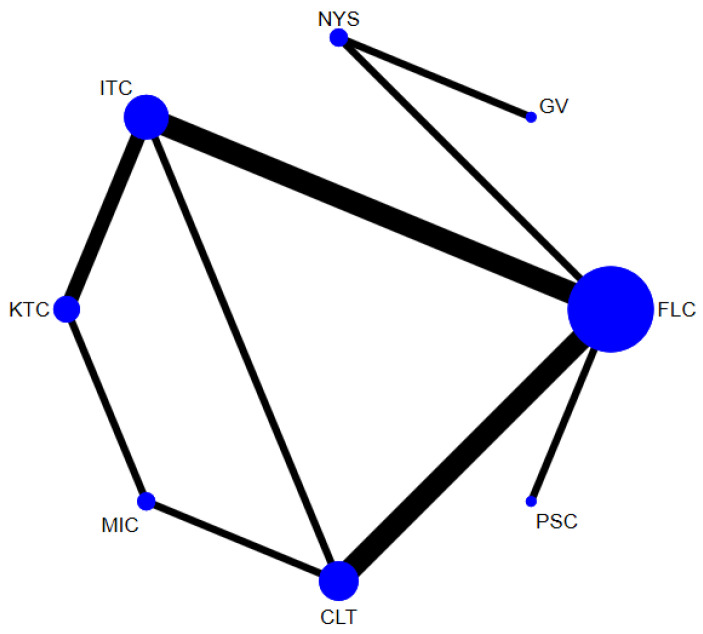
Network plot comparing the efficacy of antifungal agents used for the treatment of OPC among HIV-infected adults (clinical cure). Abbreviations: CLT—clotrimazole, FLC—fluconazole, GV—gentian violet, ITC—itraconazole, KTC—ketoconazole, MIC—miconazole, NYS—nystatin, PSC—posaconazole.

In total, 14 randomized control trials [13,14,15,16,20,40,41,42,43,45,49,50,52,53] with 2760 participants comparing 8 interventions (Figure 3) were included in this NMA, which is expressed as the risk ratio (RR) of achieving clinical cure when treated with the specified antifungal agent as compared to fluconazole. When assessing the comparative efficacy of different antifungal agents with fluconazole, no significant difference was observed between any interventions. Only two interventions were significantly less effective than fluconazole (gentian violet [RR, 0.61 (95% CI = 0.40–0.94)] and nystatin [RR 0.59 (95% CI = 0.43–0.82)]). 

**Figure 4:**
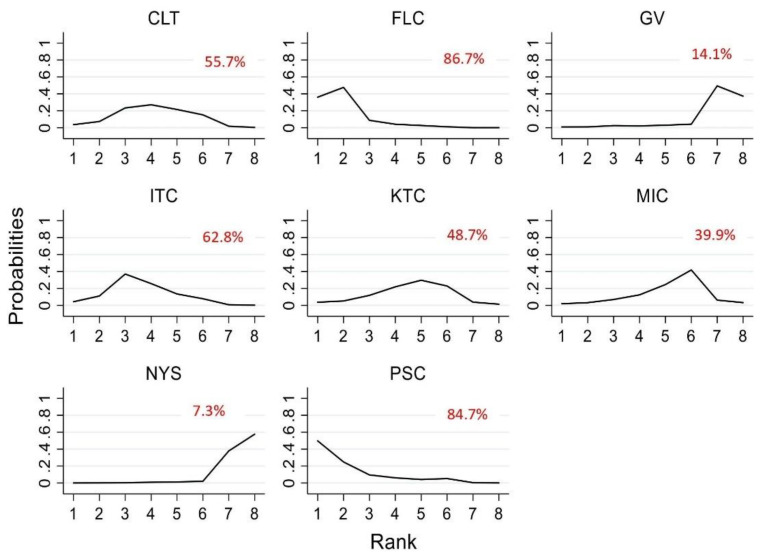
SUCRA ranking curve of antifungal agents used for the treatment of OPC among HIV-infected adults (clinical cure). Abbreviations: CLT—clotrimazole, FLC—fluconazole, GV—gentian violet, ITC—itraconazole, KTC—ketoconazole, MIC—miconazole, NYS—nystatin, PSC—posaconazole. *X*-axis: ranking of treatment. *Y*-axis: probability of a given treatment to be the first, second, third, or fourth best. In this example, treatment A has the largest probability to be the first best treatment.

**Figure 5:**
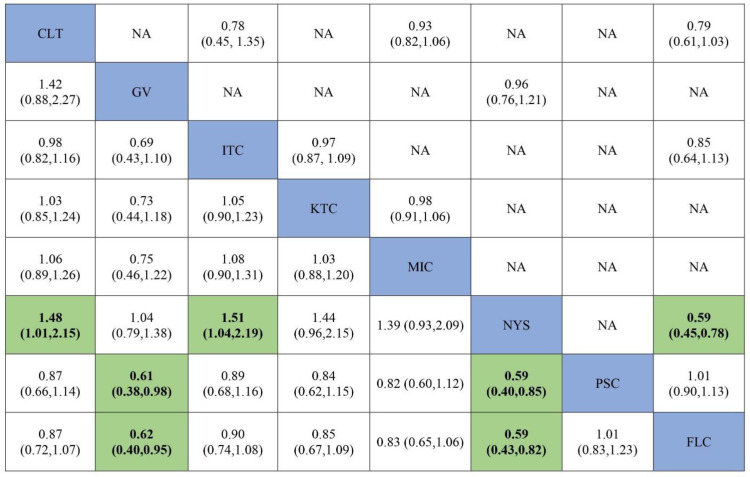
Comparative efficacy of antifungal agents used for the treatment of OPC among HIV-infected adults (clinical cure). Note: pairwise (upper right portion) and network (lower left portion) meta-analytic results. Outcomes are expressed as risk ratio (95% confidence intervals). For the pairwise meta-analyses, a relative risk of more than 1 indicates that the treatment specified in the row is more efficient. For the network meta-analysis, a relative risk of more than 1 shows that the treatment specified in the column is more efficient. Bold and green shaded results indicate statistical significance. NA—no direct comparison to show the effect size. Abbreviations: CLT—clotrimazole, FLC—fluconazole, GV—gentian violet, ITC—itraconazole, KTC—ketoconazole, MIC—miconazole, NYS—nystatin, PSC—posaconazole.

The ranking of the efficacy was based on SUCRA, and fluconazole appears to be the most efficacious antifungal followed by posaconazole, itraconazole, clotrimazole and ketoconazole. The least effective antifungal agent was nystatin, followed by gentian violet and miconazole. Table 2 summarizes the RR and the ranking of the antifungal agents while Figure 4 shows the SUCRA ranking curves for each antifungal agent in the network. The direct and network estimates for the efficacy of these agents are shown in the league table, as shown in Figure 5.

### 3.5. Efficacy of Antifungal Agents Used in the Treatment of OPC to Achieve Clinical Cure (Pairwise Meta-Analysis)

There were no statistically significant findings from the pairwise meta-analysis except for the comparison between fluconazole and nystatin, where fluconazole was statistically more effective than nystatin in treating OPC among HIV-infected adults (RR, 0.59 (95% CI = 0.45–0.78)). The results from the pairwise meta-analyses of the studies included are shown in the forest plot (Supplementary Figure S1).

### 3.6. Efficacy of Antifungal Agents Used in the Treatment of OPC to Achieve Mycological Cure (Network Meta-Analysis)

**Figure 6:**
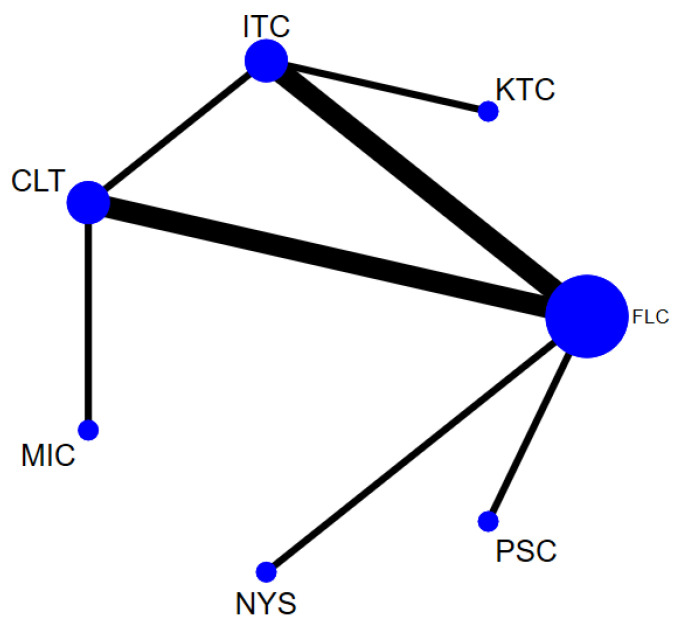
Network plot of trials comparing the efficacy of antifungal agents used for the treatment of OPC among HIV-infected adults (mycological cure). Abbreviations: CLT—clotrimazole, FLC—fluconazole, ITC—itraconazole, KTC—ketoconazole, MIC—miconazole, NYS—nystatin, PSC—posaconazole.

**Figure 7:**
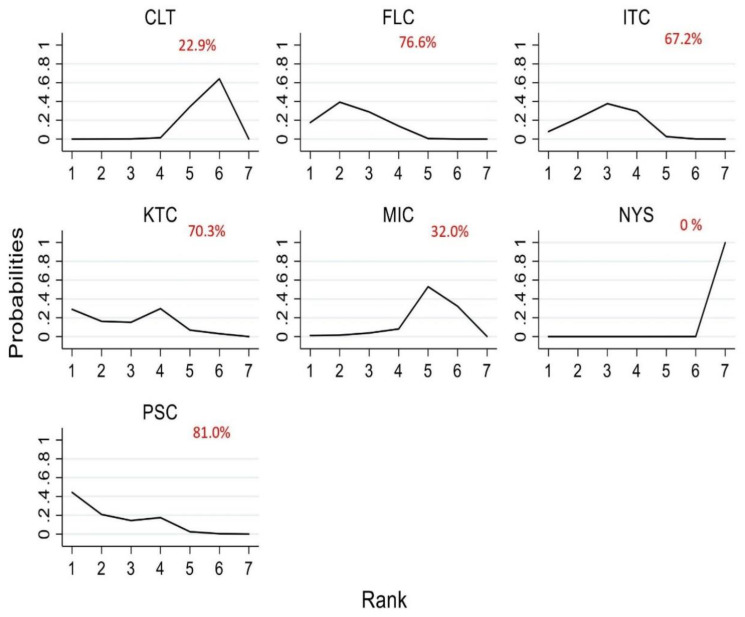
SUCRA ranking curve for the efficacy of antifungal agents used for the treatment of OPC among HIV-infected adults (mycological cure). Abbreviations: CLT—clotrimazole, FLC—fluconazole, ITC—itraconazole, KTC—ketoconazole, MIC—miconazole, NYS—nystatin, PSC—posaconazole. *X*-axis: ranking of treatment. *Y*-axis: probability of a given treatment to be the first, second, third, or fourth best. In this example, treatment A has the largest probability to be the first best treatment.

**Figure 8:**
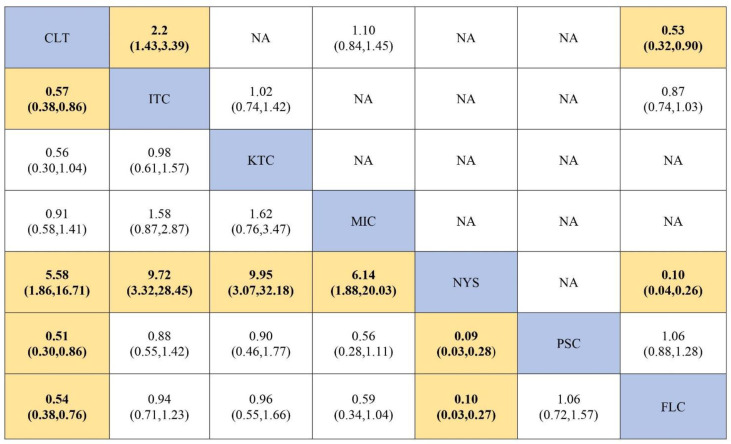
Comparative efficacy of different antifungal agents in the treatment of OPC (mycological cure). Note: pairwise (upper right portion) and network (lower left portion) meta-analytic results. Outcomes are expressed as risk ratio (95% confidence intervals). For the pairwise meta-analyses, a relative risk of more than 1 indicates that the treatment specified in the row is more efficient. For the network meta-analysis, a relative risk of more than 1 shows that the treatment specified in the column is more efficient. Bold and peach shaded results indicate statistical significance. NA—there is no direct comparison to show the effect size. Abbreviations: CLT—clotrimazole, FLC—fluconazole, ITC—itraconazole, KTC—ketoconazole, MIC—miconazole, NYS—nystatin, PSC—posaconazole.

Eleven randomized controlled trials [13,14,15,16,20,40,41,42,44,49,50] were analyzed to determine the efficacy of antifungal agents in producing a mycological cure among HIV-infected adults. The network plot derived is shown in Figure 6. In the network analysis, fluconazole was used as the reference antifungal agent. The network estimate shows that when compared to fluconazole, nystatin (RR, 0.10 (95% CI = 0.03–0.27)) was the least effective in achieving mycological cure, followed by clotrimazole (RR, 0.54 (95% CI = 0.37–0.76)) and these findings were statistically significant. Table 3 summarizes the RR and the ranking of the antifungal agents, while Figure 7 shows the SUCRA ranking curves for each antifungal agent in the network. Itraconazole (RR, 0.57 (95% CI = 0.38–0.86)), posaconazole (RR, 0.51 (95% CI = 0.30–0.86)) and fluconazole (RR, 0.54 (95% CI = 0.38–0.76)) were found to be significantly more effective than clotrimazole in achieving mycological cure. Clotrimazole (RR, 5.58 (95% CI = 1.86–16.71)), itraconazole (RR, 9.72 (95% CI = 3.32–28.45)), ketoconazole (RR, 9.95 (95% CI = 3.07–32.18)) and miconazole (RR, 6.14 (95% CI = 1.88–20,03)) were superior in fostering mycological cure compared to nystatin, and even though these estimates are statistically significant, wide confidence intervals could be noticed. The direct and network estimates for the efficacy of these agents are shown in the league table in Figure 8.

### 3.7. Efficacy of Antifungal Agents Used in the Treatment of OPC to Achieve Mycological Cure (Pairwise Meta-Analysis)

Fluconazole was more effective than clotrimazole (RR, 0.53 (95% CI = 0.32–0.90)) and nystatin (RR, 0.10 (95% CI = 0.04–0.26)) and clotrimazole was found to be more effective than itraconazole (RR, 2.20 (95% CI = 1.43–3.39)). Pairwise meta-analysis was conducted for studies with direct comparison and the forest plot for this comparison is shown in Supplementary Figure S2. 

### 3.8. Safety of Antifungal Agents Used in Treating OPC

The total number of adverse effects reported for each drug in each trial was used to analyze the safety profile. There were 36 different adverse effects identified and further grouped, under an umbrella classification, wherever possible. Effects such as nausea, vomiting, diarrhea, flatulence, gastroenteritis, abdominal pain, and dysphagia were categorized as gastrointestinal adverse effects while neurological adverse effects included dizziness, paresthesia, coma, convulsions, and hemiparesis; rashes, exanthema, pruritus, and Steven–Johnson Syndrome were considered as dermatological adverse effects; the incidence of hypotension and palpitation was classified as cardiovascular adverse events; adverse events such as cough, shortness of breath and upper respiratory tract infections were grouped as respiratory adverse events.

**Figure 9:**
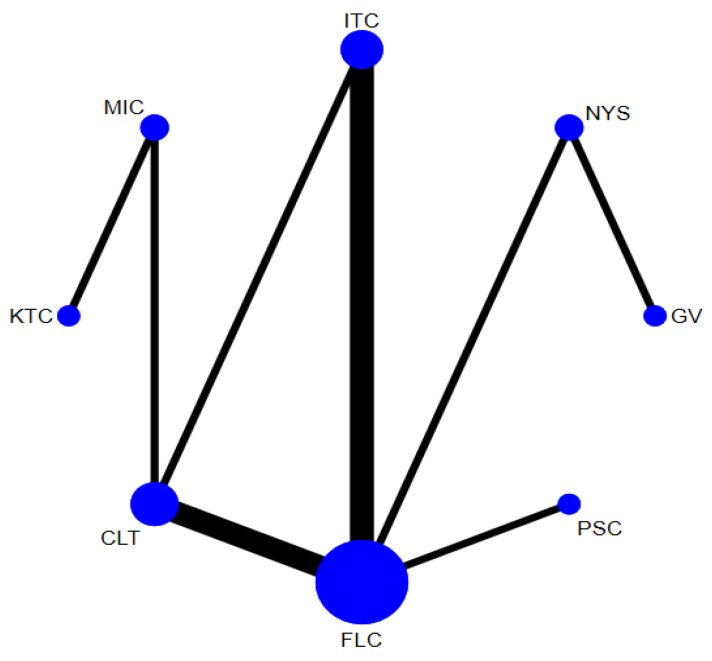
Network plot of trials comparing the safety of antifungal agents used for the treatment of OPC among HIV-infected adults. Abbreviations: CLT—clotrimazole, FLC—fluconazole, GV—gentian violet, ITC—itraconazole, KTC—ketoconazole, MIC—miconazole, NYS—nystatin, PSC—posaconazole.

**Figure 10:**
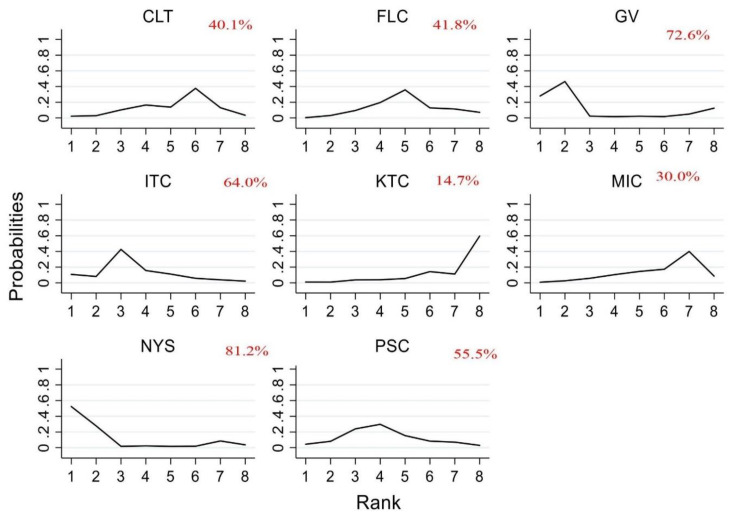
SUCRA ranking curve for the safety of antifungal agents used for the treatment of OPC among HIV-infected adults. Abbreviations: CLT—clotrimazole, FLC—fluconazole, GV—gentian violet, ITC—itraconazole, KTC—ketoconazole, MIC—miconazole, NYS—nystatin, PSC—posaconazole.

**Figure 11:**
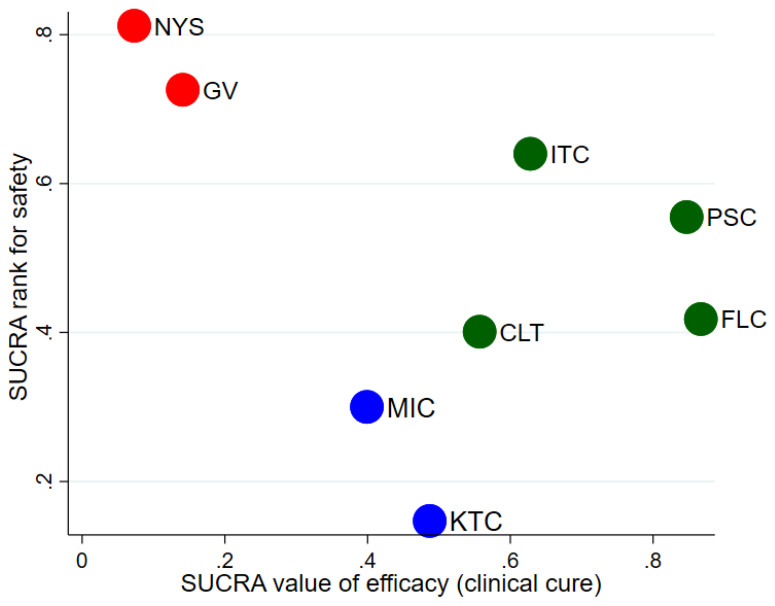
Cluster plot for SUCRA rankings for combined efficacy and safety outcomes. Notes: for efficacy outcomes, lower score indicates better treatment in preventing oral candidiasis. For safety outcomes, lower score indicates safer treatment with lower risk of adverse events. Interventions that are located at left lower site are the most effective and safest in preventing oral candidiasis. Abbreviations: CLT—clotrimazole, FLC—fluconazole, GV—gentian violet, ITC—itraconazole, KTC—ketoconazole, MIC—miconazole, NYS—nystatin, PSC—posaconazole.

The network was formed with 12 studies, as shown in Figure 9. The safety of the antifungal agents was compared against fluconazole as the reference comparator and four agents were ranked above it; in order of highest-ranking: nystatin, gentian violet, itraconazole and posaconazole. Clotrimazole, miconazole and ketoconazole were ranked lower than fluconazole. However, none of these comparisons was statistically significant (Supplementary Figures S3 and S4). The SUCRA ranking plot is shown in Figure 10 and the RR arranged in sequence from the SUCRA ranking is shown in Table 4. The cluster plot for the combined efficacy and safety outcomes based on SUCRA is provided in Figure 11. As per the cluster ranking plot, fluconozaole is more effective, with a favorable safety profile compared to other treatments, followed by posaconazole.

### 3.9. Rate of OPC Relapse upon Treatment Completion

The rate of relapse of OPC among those who received treatment for OPC was reported in 12 out of the 20 studies, which were included in the qualitative analysis. These studies reported the number of patients relapsing with OPC 14 days and 28 days after completion of treatment. Four studies reported statistically significant differences in rates of OPC relapse 14 days after completion of treatment. Koletar et al. [13], Redding et al. [14], and Pons et al. [40] reported that a significantly higher number of patients from the clotrimazole arm had OPC relapse as compared to fluconazole. Pons et al. [50] reported a significantly higher rate of relapse among those who received nystatin compared to fluconazole. The findings of this study are reported as recommended by the PRISMA extension statement of NMA (Supplementary Table S4).

### 3.10. GRADE Quality Assessment

Twenty-eight comparisons were made, three of which were of high quality, seventeen were of moderate quality and eight were of low quality (Supplementary Table S5).

### 3.11. Network Consistency and Publication Bias

There was no inconsistency shown for any outcome in the NMA (Supplementary Table S6) Based on the comparison-adjusted plots (Supplementary Figures S5–S7), publication bias could be detected. 

## 4. Discussion

As far as we are aware, currently, there is no published network meta-analysis which compared the effectiveness and safety of antifungal agents used in treating and preventing OPC among HIV-infected adults. Only systematic reviews and meta-analyses [55,56] have been published with regard to this population of interest. To the best of our knowledge, this is the first NMA performed which ranked the antifungal agents used in the treatment of OPC in HIV-infected adults in terms of their efficacy in achieving the clinical cure, mycological cure as well as their safety profile.

The efficacy analysis for the clinical cure was carried out with information gathered from fourteen RCTs involving nine comparisons of eight different antifungal agents. In our results, SUCRA ranking fluconazole was ranked first compared to all other antifungal agents. However, the NMA illustrated that other drugs, including posaconazole, itraconazole, clotrimazole, ketoconazole and miconazole, were not inferior to fluconazole in achieving the clinical cure. The efficacy estimate in terms of mycological cure was obtained from eleven RCTs comparing seven different antifungal agents. Fluconazole was ranked second after posaconazole with regard to achieving the mycological cure; however, the relative risk was close to one and this was not found to be statistically significant, hinting that both drugs could be equally effective. 

The possible reason behind fluconazole’s superiority may lie in its pharmacodynamic and pharmacokinetic properties. Compared to ketoconazole, fluconazole has a higher affinity for the cytochrome CYP450 enzyme in fungi than human cells [57], making it more effective in exerting its antifungal effects and less likely to cause drug–drug interaction due to its lack of affinity towards the human cytochrome CYP3A4 enzyme. The absorption of fluconazole, unlike itraconazole and ketoconazole, is not influenced by the presence of gastric pH [58,59,60,61], therefore increasing its bioavailability and improving its effectiveness.

The findings from our study suggested that posaconazole has similar effectiveness in achieving clinical and mycological cures in OPC among HIV patients when compared to fluconazole. However, among the selected trials, there was only one RCT that compared posaconazole with fluconazole [20]. In this study, it was reported that even though these two drugs appeared to be equally effective, patients who received posaconazole were more likely to remain disease free after completion of treatment than those who received fluconazole [20]. Posaconazole is a triazole antifungal agent and inhibits the synthesis of ergosterol by the inhibition of the enzyme, lanosterol 14-alpha demethylase, with the accumulation of methylated sterol precursors [20]. Posaconazole demonstrated antifungal activity on isolates that were found to be resistant to both itraconazole and fluconazole [62]. However, more studies would be needed to elucidate the appeared superiority of posaconazole over fluconazole in terms of achieving a longer disease-free period when used to treat OPC among HIV patients. 

Our study also illustrated that nystatin was the least effective drug in achieving clinical cure or a mycological cure of OPC among HIV adults. The second least effective drug was gentian violet in terms of achieving clinical cure, and clotrimazole in achieving the mycological cure. Furthermore, the rate of OPC relapse was significantly higher among those who received nystatin and clotrimazole when compared to fluconazole. All three of these agents are topical antifungals. Treating OPC in HIV patients with topical antifungal agents often ends with failure [10]. The reason for this includes non-compliance, as most of the topical agents require multiple dosing; for example, clotrimazole troche is required to be administered five times a day, while nystatin mouthwash needs to be administered four times a day. Both Koletar et al. [13] and Redding et al. [14] also highlighted that more patients were compliant to fluconazole, which requires a daily dose compared to clotrimazole troche. Inadequate drug concentration and duration of exposure have been identified as one of the contributing factors in the lack of effectiveness of topical antifungal agents [10]. Although topical antifungal agents are the preferred choice for uncomplicated OPC in healthy patients, they are not a choice of drug to treat HIV-infected adults due to the lack of apparent effectiveness in achieving a clinical and mycological cure. 

The results regarding the safety of the antifungal agents used in treating OPC showed that none of the antifungal agents appeared to be significantly safer than fluconazole. Gentian violet and nystatin were ranked the safest by the SUCRA graph, and this could be because as topical drugs, less systemic exposure would mean lesser chances of the patient experiencing adverse effects from it [63]. The favorable safety profile of fluconazole as compared to the other agents from the same class of agents is probably due to its weaker affinity for the CYP3A4 liver enzyme, indirectly suggesting lesser drug–drug interactions [60]. This is significant concerning HIV patients, who are usually on antiretroviral therapy, and it would be better to minimize drug–drug interaction as much as possible. A meta-analysis by Wang et al. that compared the safety profile of systemic antifungal agents reported that patients receiving itraconazole had a higher chance of treatment discontinuation due to adverse effects than those receiving fluconazole [64]. More patients receiving itraconazole were found to experience hepatotoxicity compared to those who received fluconazole, and the authors suggested that fluconazole has a better hepatic safety profile than itraconazole. Together, these findings suggest that fluconazole appears to have a better safety profile compared to the other systemic antifungal agents, and while topical agents have been ranked the safest, they lack in terms of clinical and mycological efficacy.

The emergence of drug resistance has been a major issue with the treatment of candidal infections, and monitoring resistance is vital in observing the response in a hospital setting. There is extensive documentation of resistance to azole antifungals among *Candida albicans* and other less prevalent species, including *Candida glabrata*, *Candida parapsilosis*, *Candida tropicalis* and *Candida krusei*. The incidence of reported fluconazole resistance in *C.albicans* isolated from OPC is higher, and mainly varies upon prior azole treatment as well as OPC episodes [65]. *C. glabrata* is known to exhibit intrinsic diminished susceptibility to the azole antifungals, and therefore presents with azole resistance more frequently [66]. Moreover, this organism is more frequently isolated from patients receiving fluconazole prophylaxis [67]. *C. krusei* also exhibits intrinsic resistance to azoles, and increased infection rate is related to fluconazole prophylaxis or previous treatment [68,69]. Fluconazole resistance as high as 83% was identified in *C. tropicalis* isolated from the Asia–Pacific region [70]. The incidence of fluconazole resistance worldwide in *C. parapsilosis* infections ranges between 2 and 5% [71]. Itraconazole, posaconazole, voriconazole and the latest addition to the azole family, isavuconazole, was used in such fluconazole refractory cases [72]. Amphotericin B oral suspension was recommended by IDSA as an alternative for fluconazole in refractory oral candidiasis [73]. Combining azoles with other antifungal agents was recommended to broaden the spectrum of activity to deal with azole-resistant fungi. Successful outcomes in OPC with a combination regime inclusive of azoles with amphotericin B or terbinafine were documented in case reports [74,75]. However, concrete evidence on the comparative efficacy of these drugs in fluconazole refractory cases is lacking, and further clinical trials are required. 

Another class of newer antifungals which are becoming increasingly popular for the treatment of severe candidiasis in immunodeficient patients are the echinocandins. Echinocandins were proven to be as effective and safe as fluconazole for the treatment of esophageal candidiasis in patients with HIV infection [76,77,78]. These agents have fungicidal action and may be superior in terms of clinical response and the complete resolution of oropharyngeal candidiasis in comparison with the commonly used fungistatic drug (azoles) considering (1) anatomical proximity and similarity of esophageal and oropharyngeal mucosa, and (2) echinocandins were shown to be more potent than commonly used azoles against *Candida* biofilms in vitro studies [79,80]. Thus, further clinical trials with echinocandins in the context of oropharyngeal candidiasis in HIV patients are warranted, owing to its potential beneficial role in the treatment of OPC infections, especially those that do not respond to azoles.

There were a few limitations in this study. Firstly, most of the comparison arms in our network had one trial connecting them; more studies would lead to more precise and accurate estimates. Some of the studies included in this study had mostly male participants. For example, the study by Smith et al. [43] and Redding et al. [14] had no female participants in their study population. Moreover, the doses of fluconazole and itraconazole varied among the studies analyzed and ranged from 100–200 mg. Thus, variability in the doses of interventions could not be accounted for in our study, as there were limited data to perform individual analysis on the dose–response effect for each intervention. Finally, there are some inconsistencies in the definition of adverse events across trials. Hence, data had to be pooled for the analysis of adverse effects, and some of the adverse effects would have had more influence on the results, regardless of the severity.

## 5. Conclusions

The findings from our NMA illustrate that among the trials conducted exclusively for the treatment of OPC, fluconazole was ranked the most effective antifungal agent and has reasonable safety. However, the possibility of resistance must be accounted for, and hence, further trials with newer fungicidal agents are warranted.

## Figures and Tables

**Table 1:** Characteristics of the included studied in detail.

Author, Year	Country	No. of Randomized Participants	Study Design	Study Comparison	Gender	
					Male	Female
Koletar SL et al., 1990	NM	36	RCT	Fluconazole ^s^ 100 mg OD vs. Clotrimazole ^t^ 10 mg 5× Daily day	33	3
Smith DE et al., 1991	UK	85	RCT	Itraconazole ^s^ 200 mg OD vs. Ketoconazole ^s^ 200 mg BD	85	0
Redding S.W. et al., 1992	US	24	RCT	Fluconazole ^s^ 100 mg OD vs. Clotrimazole ^t^ 10 mg 5× Daily	24	0
De Wit S et al., 1993	NM	56	RCT	Single dose Fluconazole ^s^ 150 mg vs. Daily Fluconazole ^s^ 50 mg	NM	NM
Pons V et al., 1993	NM	334	RCT	Fluconazole ^s^ 100 mg OD vs. Clotrimazole ^t^ 10 mg 5× a day	308	26
de Repentigny L et al., 1996	Canada	106	RCT	Itraconazole ^s^ 200 mg OD vs. Ketoconazole ^s^ 200 mg OD	93	5
De Wit S et al., 1997	Belgium, UK, France	27	RCT	D0870 ^s^(100 mg/25 mg) vs. D0870 ^s^ (10 mg/10 mg)	23	4
Murray PA et al., 1997	US	162	RCT	Itraconazole ^s^ 200 mg OD vs. Clotrimazole ^t^ 10 mg 5× a day	120	29
Pons V et al., 1997	US	167	RCT	Fluconazole ^s^ 100 mg OD vs. Nystatin ^t^ 500,000 U QID	NM	NM
De Wit S et al., 1998	Belgium	40	RCT	Single dose Fluconazole ^s^ 150 mg vs. Itraconazole ^s^ 100 mg OD	32	8
Graybill JR et al., 1998	US	190	RCT	Itraconazole ^s^ 200 mg OD vs. Fluconazole ^s^ 100 mg OD	166	13
Phillips P et al., 1998	Austria, Belgium, Canada, Germany, Netherlands, Spain, UK	244	RCT	Itraconazole ^s^ 100 mg OD/BD vs. Fluconazole ^s^ 100 mg OD	221	23
Linpiyawan R et al., 2000	Thailand	29	RCT	Clotrimazole ^t^ 10 mg 5× Daily vs. Itraconazole ^s^ 100 mg BD	20	9
Vazquez JA et al., 2002	US	25	RCT	Alcohol-based Melaleuca ^t^ 15 mL QID vs. Alcohol-free Melaleuca ^t^ 5 mL QID	25	0
Van Roey J et al., 2004	Uganda	357	RCT	Miconazole ^t^ 10 mg OD vs. Ketoconazole ^s^ 400 mg OD	82	275
Vazquez JA et al., 2006	US, Europe, Latin America, Canada, South Africa	350	RCT	Posaconazole ^s^ 200 mg Day 1, 100 mg OD vs. Fluconazole ^s^ 200 mg Day 1, 100 mg OD	262	88
Hamza OJM et al., 2008	Tanzania	220	RCT	Single dose Fluconazole ^s^ 750 mg vs. Daily Fluconazole ^s^ 150 mg OD	53	167
Wright S.C. et al., 2009	South Africa	90	RCT	Gentian violet ^t^ 0.5% TDS vs. Lemon juice ^t^ TDS vs. Lemongrass ^t^ BD	22	60
Vazquez JA et al., 2010	US, Canada, South Africa	578	RCT	Miconazole ^t^ 10 mg OD vs. Clotrimazole ^t^ 10 mg 5× Daily	236	341
Mukherjee PK et al., 2017	South Africa, India, Uganda, Kenya, Botswana, Malawi, Zimbabwe	221	RCT	Gentian violet ^t^ 0.00165% BD vs. Nystatin ^t^ 500,000 U QID	93	128

Abbreviations: NM, not mentioned; RCT, randomized control trial, OD, once daily, BD, twice a day, TDS, three times a day, QID, four times a day, ^t^ topical, ^s^ systemic.

**Table 2:** Network estimates and SUCRA ranking of the efficacy of antifungal agents used for the treatment of OPC among HIV-infected adults (clinical cure).

Intervention	All RCTs		
	RR [95% CI]	*p*-Value	SUCRA Rank
Fluconazole	Reference		1
Posaconazole	1.01 (0.83,1.23)	0.91	2
Itraconazole	0.89 (0.74,1.08)	0.25	3
Clotrimazole	0.87 (0.7,1.06)	0.19	4
Ketoconazole	0.85 (0.67,1.09)	0.20	5
Miconazole	0.82 (0.64,1.05)	0.13	6
Gentian violet	0.61 (0.40,0.94)	0.02	7
Nystatin	0.59 (0.43,0.82)	0.001	8
Overall inconsistency Chi-square (*p* value)	0.92 (0.6312)		
Number of studies	14		

**Table 3:** NMA estimates and SUCRA ranking of antifungal agents used for the treatment of OPC among HIV-infected adults (mycological cure).

Intervention	All RCTs		
	RR [95% CI]	*p*-Value	SUCRA Rank
Posaconazole	1.06 (0.71–1.56)	0.77	1
Fluconazole	Reference		2
Ketoconazole	0.95 (0.55–1.65)	0.88	3
Itraconazole	0.93 (0.71–1.22)	0.64	4
Miconazole	0.59 (0.34–1.04)	0.06	5
Clotrimazole	0.54 (0.37–0.76)	0.001	6
Nystatin	0.10 (0.03–0.27)	0.00	7
Overall inconsistency Chi-square (*p* value)	3.35(0.674)		
Number of studies	11		

**Table 4:** Network estimates and SUCRA ranking of the safety of antifungal agents used for the treatment of OPC among HIV-infected adults.

Intervention	All RCTs		
	RR [95% CI]	*p*-Value	SUCRA Rank
Nystatin	0.33 (0.03,3.10)	0.33	1
Gentian violet	0.38 (0.04,3.94)	0.42	2
Itraconazole	0.89 (0.71,1.12)	0.30	3
Posaconazole	0.94 (0.81,1.09)	0.43	4
Fluconazole	Reference		5
Clotrimazole	1.07 (0.72,1.61)	0.22	6
Miconazole	1.12 (0.73,1.73)	0.59	7
Ketoconazole	1.29 (0.76,2.20)	0.34	8
Overall inconsistency Chi-square (*p* value)	1.57 (0.2106)		
Number of studies	12		

## Supplementary Materials

The following are available online at https://www.mdpi.com/article/10.3390/jof7080637/s1, Figure S1: Forest plot of pairwise meta-analysis comparing antifungal agents used for the treatment of OPC among HIV-infected adults (clinical cure), Figure S2: Forest plot of pairwise meta-analysis antifungal agents used for the treatment of OPC among HIV-infected adults (mycological cure), Figure S3: Comparative safety of different antifungal agents in treating OPC, Figure S4: Forest plot of pairwise meta-analysis of the safety of antifungal agents used for the treatment of OPC among HIV-infected adults, Figure S5: Comparison-adjusted funnel plot of interventions used for the treatment of OPC among HIV-infected adults (clinical cure), Figure S6: Comparison-adjusted funnel plot of interventions used for treatment of OPC among HIV-infected adults (mycological cure), Figure S7: Comparison-adjusted funnel plot of interventions used for the treatment of OPC among HIV-infected adults (safety profile), Table S1: Search strategy, Table S2: Search algorithm for Medline, Embase, CENTRAL and Scopus, Table S3: Studies excluded with reason from full text screening, Table S4: NMA PRISMA Checklist, Table S5: GRADE comparison of antifungal agents used in treating OPC, Table S6: Global inconsistency in networks using the ‘design-by-treatment’ interaction mode.
