# Supplementary material for: Comparative Efficacy of Antifungal Agents Used in the Treatment of Oropharyngeal Candidiasis among HIV-Infected Adults: A Systematic Review and Network Meta-Analysis
Source: J Fungi (Basel). 2021 Aug 5;7(8):637. doi: 10.3390/jof7080637 (PMC8398874; doi:10.3390/jof7080637)
Supplement: Supplementary file 1 [file jof-07-00637-s001.zip › jof-1306646-supplementary.pdf]

Table S1. Search strategy.

Review question	In HIV-infected adults, which antifungal treatment is more effective and safer to treat OPC?
**Population**	HIV-infected adults receiving treatment for OPC
**Sub-group**	If heterogenicity is present:<br>1. Drug doses<br>2. Dosing frequency
**Intervention**	Any intervention which is meant to treat OPC, including systemic and topical antifungal agents, traditional medication, and other interventions.
**Comparison**	Placebo, no treatment, or any intervention against those interventions mentioned above (including different doses of the same intervention).
**Outcomes**	***Primary outcome:*** presence or absence of clinical oropharyngeal candidiasis<br>***Secondary outcome:*** mycological cure of OPC, relief of pain, relief of dysphagia, incidence of systemic infection, use of empirical antifungal treatment, adverse events ('probably due to drug'), compliance, development of drug resistance or time to relapse after completing the treatment if mentioned by the study.
**Study design**	RCTs or Systematic reviews
**Databases**	Medline, Scopus, Embase, CENTRAL

Table S2. Search algorithm for Medline, Embase, CENTRAL and Scopus.

Database	Query		Items found
**Medline on Ovid**	1. HIV	exp HIV/ or HIV.mp. OR HIV Infections.mp. or exp HIV Infections/ OR hiv-1.mp. or exp HIV-1/ OR hiv-2.mp. or exp HIV-2/ OR human immunodeficiency virus.mp. or exp HIV/ OR human immune-deficient virus.mp. OR (human immun*.mp. AND deficiency virus.mp.) OR exp Acquired Immunodeficiency Syndrome/ or acquired deficiency virus.mp. OR acquired immune-deficiency.mp. or exp AIDS-Related Opportunistic Infections/ OR AIDS.mp. or exp Acquired Immunodeficiency Syndrome/ OR acquired immun*.mp. OR deficiency syndrome.mp. OR sexually transmitted diseases, viral/ or exp hiv infections/	463999
	2. Candidiasis	candidiasis.mp. OR exp Candidiasis, Chronic Mucocutaneous/ or Candidiasis/ or exp Candidiasis, Oral/ OR thrush.mp. OR candidiosis.mp. OR candida infect*.mp. OR oral candidiasis.mp. OR candidi*.mp. OR Candida/ or candida.mp.	87375
	3. Oropharyngeal	oropharyngeal.mp. OR oral disease*.mp. OR orophyarynx.mp.	23688
	4. Randomized control trials	exp Randomized Controlled Trials as Topic/ OR Clinical Trials as Topic/ or Double-Blind Method/ or controlled clinical trial*.mp. or Randomized Controlled Trials as Topic/ OR clinical trial.mp. or Clinical Trial/ OR random allocation.mp. or *Random Allocation/ OR single blind method.mp. or Single-Blind Method/ OR research design.mp. OR comparative stud*.mp. OR *Prospective Studies/ or prospective stud*.mp. OR exp Evaluation Studies as Topic/ or evaluation stud*.mp.	4243433
	5.	# 2 AND #3	1398
	6.	#1 AND #5	608
	7.	#4 AND #6	**251**
**Embase on Ovid**	1. HIV	HIV.mp. or exp Human immunodeficiency virus/ OR HIV infections.mp. or Human immunodeficiency virus infection/ OR hiv-1.mp. or exp Human immunodeficiency virus 1/ OR hiv-2.mp. or exp Human immunodeficiency virus 2/ OR exp Human immunodeficiency virus/ or human immunodeficiency virus.mp. OR human immune deficient virus.mp. OR (human immun*.mp. AND deficiency virus.mp.) OR exp acquired immune deficiency syndrome/ or acquired immunodeficiency virus.mp. or exp Human immunodeficiency virus infection/ OR acquired immune deficiency.mp. OR AIDS.mp. or exp acquired immune deficiency syndrome/ OR (acquired immun*.mp. AND deficiency syndrome.mp.) OR sexually transmitted diseases,viral.mp. or sexually transmitted disease/	636468
	2. Candidiasis	candidiasis, oral.mp. or exp thrush/ OR candidiosis.mp. OR candida infect*.mp.<br>OR candidi*.mp. OR exp Candida/ or candida.mp.	140492
	3. Oropharyngeal	oropharyngeal.mp. OR oral disease.mp. or exp mouth disease/ OR oropharynx.mp. or exp oropharynx/ or exp oropharynx candidiasis	650314
	4. Randomized control trial	Randomized Controlled Trial.mp. or exp randomized controlled trial/ OR clinical trial.mp. or exp clinical trial/ OR double blind procedure/ or double blind.mp. OR controlled clinical trial.mp. or exp controlled clinical trial/ OR<br>single blind.mp. or exp single blind procedure/ OR random allocation.mp. or exp randomization/ OR exp placebo/ or placebo.mp. OR research design.mp. or exp methodology/ OR exp comparative study/ or comparative stud*.mp. OR exp prospective study/ or prospective stud*.mp. OR exp evaluation study/ or evaluation stud*.mp.	8225167
	5.	#2 AND #3	15753
	6.	#1 AND #5	3719
	7.	# 4 AND #6	**1071**
**Scopus**		(((TITLE-ABS-KEY ("HIV" OR "hiv-1" OR "hiv-2" OR "human immunodeficiency virus" OR "human immune deficiency virus" OR "human immune-deficiency virus" OR "HIV infections")) OR (TITLE-ABS-KEY ("acquired immune deficiency" OR "acquired immune deficiency syndrome" OR "acquired immunodeficiency syndrome" OR "acquired immunodeficiency" OR "AIDS" OR	**136**

		"AIDS related complex" OR "AIDS defining illnes*" OR "opportunistic infection*"))) AND (TITLE-ABS-KEY ("candidiasis" OR " candidiosis" OR "candida" OR "candidiasis infect*" OR "candidiosis infect*" OR "thrush" OR "oral candidiasis" OR "candidi*")) AND (TITLE-ABS-KEY ("oropharyngeal" OR "oropharynx" OR "oral lesion" OR "oral manifestation" OR "oral diseas*"))) AND (TITLE-ABS-KEY ("randomised control trial*" OR "randomized control trial*" OR "double blin*" OR "single blin*" OR "comparative" OR "placebo"))	
**CENTRAL**	1. HIV	Human immunodeficiency virus.mp. or exp HIV/ OR acquired immunodeficiency syndrome/ or aids-related complex/ or aids-related opportunistic infections/ OR AIDS.mp. OR HIV Infections/ or AIDS-Related Opportunistic Infections/ or Candidiasis, Oral/	26936
	2. Oropharyngeal	oral lesion*.mp. OR oropharynx.mp. or Oropharynx/ OR oropharyngeal.mp. OR oral manifestation*.mp.	4554
	3. Candidiasis	candidiasis.mp. or Candidiasis, Oral/ or Candidiasis OR Candida/ or candida infect*.mp.	2628
	4.	#1 AND #2 AND #3	**103**

Table S3. Studies excluded with reason from full text screening.

No	Author, Year	Title	Reason for rejection
1	Arathoon et al, 2002	Randomized, Double-Blind, Multicenter Study of Caspofungin versus Amphotericin B for Treatment of Oropharyngeal and Esophageal Candidiases	Ineligible outcome (Outcome combined HIV and non-HIV patients)
2	Chavanet et al, 1992	Trial of glucose versus fat emulsion in preparation of amphotericin for use in HIV infected patients with candidiasis.	Ineligible outcome
3	De Wit S et al, 1989	Comparison of fluconazole and ketoconazole for oropharyngeal candidiasis in AIDS	Ineligible outcome (episodes)
4	Goldman M et al, 2005	A randomized study of the use of fluconazole in continuous versus episodic therapy in patients with advanced HIV infection and a history of oropharyngeal candidiasis: AIDS Clinical Trials Group Study 323/Mycoses Study Group Study 40	Ineligible outcome (episodes)
5	MacPhail LA et al, 1996	Prophylaxis with nystatin pastilles for HIV-associated oral candidiasis.	Ineligible outcome (Outcome expressed as hazard ratio)
6	Nittayananta W et al, 2008	A randomized clinical trial of chlorhexidine in the maintenance of oral candidiasis-free period in HIV infection	Ineligible outcome (Outcome expressed as time to develop recurrent OPC)
7	Nyst MJ et al, 1992	Gentian violet, ketoconazole and nystatin in oropharyngeal and esophageal candidiasis in Zairian AIDS patients	Ineligible population (outcome of oropharyngeal lesions combined oral & esophageal lesions)
8	Scwingel A.R et al, 2012	Antimicrobial photodynamic therapy in the treatment of oral candidiasis in HIV-infected patients	Ineligible outcome (Outcome expressed as clinical signs)
9	Skiest DJ et al, 2007	Posaconazole for the treatment of azole-refractory oropharyngeal and esophageal candidiasis in subjects with HIV infection	Ineligible population (OPC & EC combined)

Table S4. NMA PRISMA Checklist.

Section/Topic	Item #	Checklist Item	Reported on Page #
**TITLE**			
Title	1	Identify the report as a systematic review *incorporating a network meta-analysis (or related form of meta-analysis).*	***1***
**ABSTRACT**			
Structured summary	2	Provide a structured summary including, as applicable:<br>**Background:** main objectives<br>**Methods:** data sources; study eligibility criteria, participants, and interventions; study appraisal; and *synthesis methods, such as network meta-analysis.*<br>**Results:** number of studies and participants identified; summary estimates with corresponding confidence/credible intervals; *treatment rankings may also be discussed. Authors may choose to summarize pairwise comparisons against a chosen treatment included in their analyses for brevity.*<br>**Discussion/Conclusions:** limitations; conclusions and implications of findings.<br>**Other:** primary source of funding; systematic review registration number with registry name.	***2***
**INTRODUCTION**			
Rationale	3	Describe the rationale for the review in the context of what is already known, *including mention of why a network meta-analysis has been conducted.*_	***4***
Objectives	4	Provide an explicit statement of questions being addressed, with reference to participants, interventions, comparisons, outcomes, and study design (PICOS).	***4,S1***
**METHODS**			
Protocol and registration	5	Indicate whether a review protocol exists and if and where it can be accessed (e.g., Web address); and, if available, provide registration information, including registration number.	***4***
Eligibility criteria	6	Specify study characteristics (e.g., PICOS, length of follow-up) and report characteristics (e.g., years considered, language, publication status) used as criteria for eligibility, giving rationale. *Clearly describe eligible treatments included in the treatment network, and note whether any have been clustered or merged into the same node (with justification).*_	***S1, 4-5***
Information sources	7	Describe all information sources (e.g., databases with dates of coverage, contact with study authors to identify additional studies) in the search and date last searched.	***4***
Search	8	Present full electronic search strategy for at least one database, including any limits used, such that it could be repeated.	***S2***
Study selection	9	State the process for selecting studies (i.e., screening, eligibility, included in systematic review, and, if applicable, included in the meta-analysis).	***6***
Data collection process	10	Describe method of data extraction from reports (e.g., piloted forms, independently, in duplicate) and any processes for obtaining and confirming data from investigators.	***5***
Data items	11	List and define all variables for which data were sought (e.g., PICOS, funding sources) and any assumptions and simplifications made.	***5,S1***
**Geometry of the network**	**S1**	Describe methods used to explore the geometry of the treatment network under study and potential biases related to it. This should include how the evidence base has been graphically summarized for presentation, and what characteristics were compiled and used to describe the evidence base to readers.	***5***
Risk of bias within individual studies	12	Describe methods used for assessing risk of bias of individual studies (including specification of whether this was done at the study or outcome level), and how this information is to be used in any data synthesis.	***5***

Summary measures	13	State the principal summary measures (e.g., risk ratio, difference in means). *Also describe the use of additional summary measures assessed, such as treatment rankings and surface under the cumulative ranking curve (SUCRA) values, as well as modified approaches used to present summary findings from meta-analyses.*	***6***
Planned methods of analysis	14	Describe the methods of handling data and combining results of studies for each network meta-analysis. This should include, but not be limited to:<br>• *Handling of multi-arm trials;*<br>• *Selection of variance structure;*<br>• *Selection of prior distributions in Bayesian analyses; and*<br>• *Assessment of model fit.*	***5-6***
**Assessment of Inconsistency**	**S2**	Describe the statistical methods used to evaluate the agreement of direct and indirect evidence in the treatment network(s) studied. Describe efforts taken to address its presence when found.	***5-6***
Risk of bias across studies	15	Specify any assessment of risk of bias that may affect the cumulative evidence (e.g., publication bias, selective reporting within studies).	***5***
Additional analyses	16	Describe methods of additional analyses if done, indicating which were pre-specified. This may include, but not be limited to, the following:<br>• Sensitivity or subgroup analyses;<br>• Meta-regression analyses;<br>• *Alternative formulations of the treatment network; and*<br>• *Use of alternative prior distributions for Bayesian analyses (if applicable).*_	***5-6***
**RESULTS†**			
Study selection	17	Give numbers of studies screened, assessed for eligibility, and included in the review, with reasons for exclusions at each stage, ideally with a flow diagram.	***6-11***
**Presentation of network structure**	**S3**	Provide a network graph of the included studies to enable visualization of the geometry of the treatment network.	***Figure 3***
**Summary of network geometry**	**S4**	Provide a brief overview of characteristics of the treatment network. This may include commentary on the abundance of trials and randomized patients for the different interventions and pairwise comparisons in the network, gaps of evidence in the treatment network, and potential biases reflected by the network structure.	***8-10***
Study characteristics	18	For each study, present characteristics for which data were extracted (e.g., study size, PICOS, follow-up period) and provide the citations.	***7***
Risk of bias within studies	19	Present data on risk of bias of each study and, if available, any outcome level assessment.	***8***
Results of individual studies	20	For all outcomes considered (benefits or harms), present, for each study: 1) simple summary data for each intervention group, and 2) effect estimates and confidence intervals. *Modified approaches may be needed to deal with information from larger networks.*	***7<br>Table 1***
Synthesis of results	21	Present results of each meta-analysis done, including confidence/credible intervals. *In larger networks, authors may focus on comparisons versus a particular comparator (e.g. placebo or standard care), with full findings presented in an appendix. League tables and forest plots may be considered to summarize pairwise comparisons.* If additional summary measures were explored (such as treatment rankings), these should also be presented.	***8-11***
**Exploration for inconsistency**	**S5**	Describe results from investigations of inconsistency. This may include such information as measures of model fit to compare consistency and inconsistency models, *P* values from statistical tests, or summary of inconsistency estimates from different parts of the treatment network.	***11***
Risk of bias across studies	22	Present results of any assessment of risk of bias across studies for the evidence base being studied.	***11***
Results of additional analyses	23	Give results of additional analyses, if done (e.g., sensitivity or subgroup analyses, meta-regression analyses, *alternative network geometries studied, alternative choice of prior distributions for Bayesian analyses,* and so forth).	***7-11<br>Supplementary material***
**DISCUSSION**			

Summary of evidence	24	Summarize the main findings, including the strength of evidence for each main outcome; consider their relevance to key groups (e.g., healthcare providers, users, and policy-makers).	***11-***
Limitations	25	Discuss limitations at study and outcome level (e.g., risk of bias), and at review level (e.g., incomplete retrieval of identified research, reporting bias). *Comment on the validity of the assumptions, such as transitivity and consistency. Comment on any concerns regarding network geometry (e.g., avoidance of certain comparisons).*	***13***
Conclusions	26	Provide a general interpretation of the results in the context of other evidence, and implications for future research.	***14***
**FUNDING**			
Funding	27	Describe sources of funding for the systematic review and other support (e.g., supply of data); role of funders for the systematic review. This should also include information regarding whether funding has been received from manufacturers of treatments in the network and/or whether some of the authors are content experts with professional conflicts of interest that could affect use of treatments in the network.	***15***

Table S5. GRADE comparison of antifungal agents used in treating OPC.

Comparison	Direct evidence		Indirect evidence		Network meta-analysis	
	Risk Ratio (95% CI)	Quality of evidence	Risk Ratio (95% CI)	Quality of evidence	Risk Ratio (95% CI)	Quality of evidence
**CLT vs FLC**	0.79(0.61,1.03)	Moderate *	0.98(0.09,10.8)	Low *ł	0.87(0.72,1.07)	Moderate
**CLT vs GV**	-	-	1.42(0.88,2.27)	Moderate₽	1.42(0.88,2.27)	Moderate
**CLT vs ITC**	0.78(0.45, 1.35)	Moderate ł	0.94 (0.11,8.34)	Low **	0.98(0.82,1.16)	Moderate
**CLT vs KTC**	-	-	1.03(0.85,1.24)	Moderate₽	1.03(0.85,1.24)	Moderate
**CLT vs MIC**	0.93(0.82,1.06)	High	1.06 (0.08,13.56)	Moderate₽	1.06 (0.89,1.26)	High
**CLT vs NYS**	-	-	1.48(1.01,2.15)	Moderate₽	1.48(1.01,2.15)	Moderate
**CLT vs PSC**	-	-	0.87(0.66,1.14)	Moderate₽	0.87(0.66,1.14)	Moderate
**GV vs FLC**	-	-	0.62(0.40,0.95)	Moderate₽	0.62(0.40,0.95)	Moderate
**GV vs ITC**	-	-	0.69(0.43,1.10)	Low **	0.69(0.43,1.10)	Low
**GV vs KTC**	-	-	0.73(0.44,1.18)	Low **	0.73(0.44,1.18)	Low
**GV vs MIC**	-	-	0.75(0.46,1.22)	Moderate₽	0.75(0.46,1.22)	Moderate
**GV vs NYS**	0.96(0.76,1.21)	High	Not estimatableŦ	Not estimatableŦ	1.04(0.79,1.38)	High
**GV vs PSC**	-	-	0.61(0.38,0.98)	Moderate₽	0.61(0.38,0.98)	Moderate
**ITC vs FLC**	0.85(0.64,1.13)	Low*Ᵽ	1.33 (0.11,15.95)	Low **	0.90(0.74,1.08)	Low
**ITC vs KTC**	0.97(0.87, 1.09)	Moderate*	1.07 (0.08,14.21)	Moderate₽	1.05(0.90,1.23)	Moderate
**ITC vs MIC**	-	-	1.08(0.90,1.31)	Moderate₽	1.08(0.90,1.31)	Moderate
**ITC vs NYS**	-	-	1.51(1.04,2.19)	Low **	1.51(1.04,2.19)	Low
**ITC vs PSC**	-	-	0.89(0.68,1.16)	Low **	0.89(0.68,1.16)	Low
**KTC vs FLC**	-	-	0.85(0.67,1.09)	Low **	0.85(0.67,1.09)	Low
**KTC vs MIC**	0.98(0.91,1.06)	Moderate*	1.03(0.08,13.42)	Moderate₽	1.03(0.88,1.20)	Moderate
**KTC vs NYS**	-	-	1.44(0.96,2.15)	Low **	1.44(0.96,2.15)	Low
**KTC vs PSC**	-	-	0.84(0.62,1.15)	Low **	0.84(0.62,1.15)	Low
**MIC vs FLC**	-	-	0.83(0.65,1.06)	Moderate₽	0.83(0.65,1.06)	Moderate
**MIC vs NYS**	-	-	1.39(0.93,2.09)	Moderate₽	1.39(0.93,2.09)	Moderate
**MIC vs PSC**	-	-	0.82(0.60,1.12)	Moderate₽	0.82(0.60,1.12)	Moderate
**NYS vs FLC**	0.59(0.45,0.78)	Moderate*	Not estimatableŦ	Not estimatableŦ	0.59(0.43,0.82)	Moderate
**NYS vs PSC**	-	-	0.59(0.40,0.85)	Moderate₽	0.59(0.40,0.85)	Moderate
**PSC vs FLC**	1.01(0.90,1.13)	High	Not estimatableŦ	Not estimatableŦ	1.01(0.83,1.23)	High

*Limitation (risk of bias). ł Imprecision. Ᵽ Publication bias suspected. ₽ Contributing direct evidence of moderate quality. ** Contributing direct evidence of low quality. Ŧ Can't be estimated as the drug was not connected in a loop in the evidence network.

Abbreviations: CLT -Clotrimazole; FLC – Fluconazole; GV – Gentian violet; ITC – Itraconazole; KTC – Ketoconazole; MIC – Miconazole; NYS – Nystatin; PSC - Posaconazole.

Table S6. Global inconsistency in networks using the 'design-by-treatment' interaction mode.

Network outcome	Chi-square	P value for test of global inconsistency
Clinical cure	0.92	0.63
Mycological cure	3.35	0.07
Adverse effects	1.57	0.21

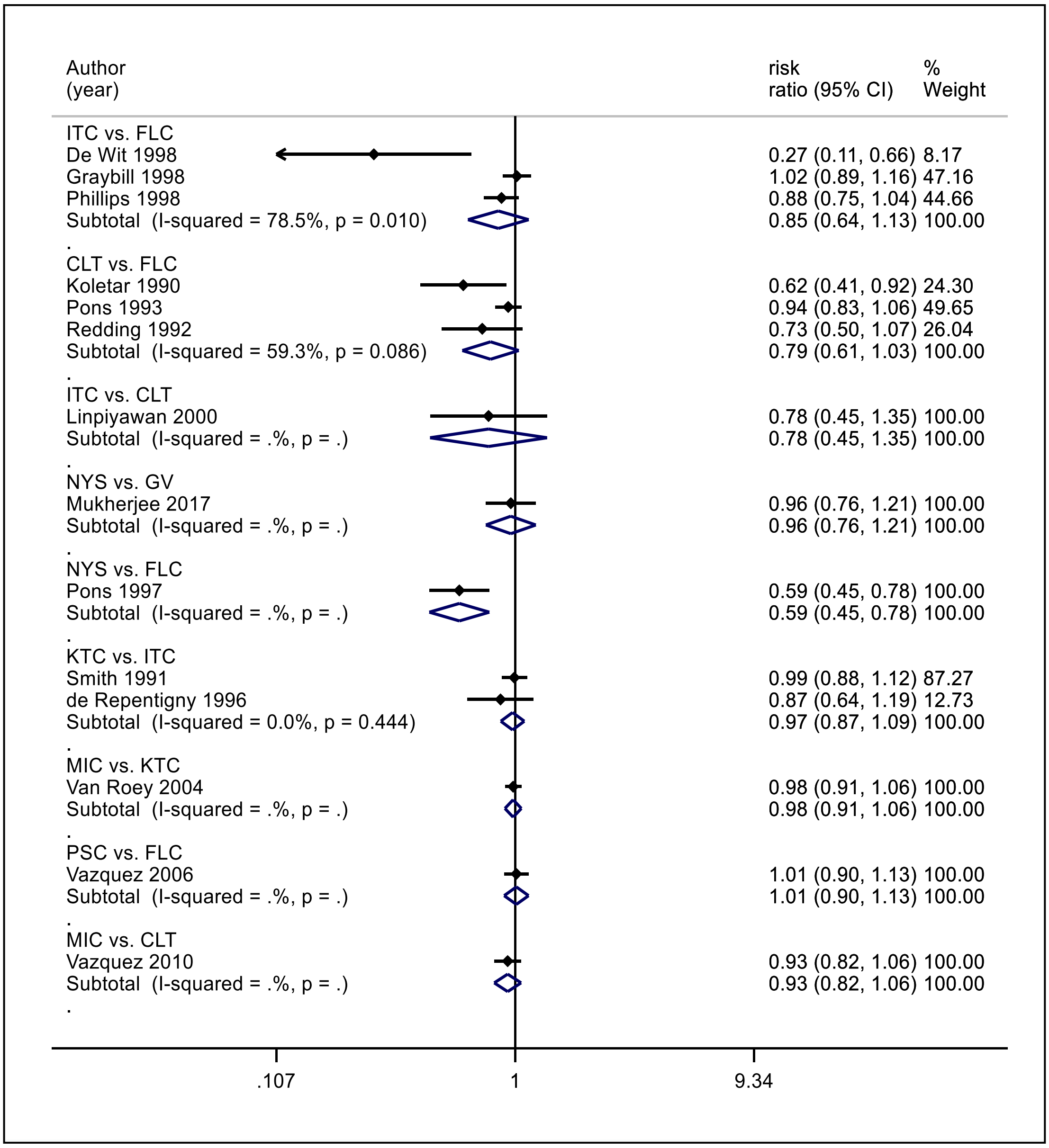

Figure S1. Forest plot of pairwise meta-analysis comparing antifungal agents used for the treatment of OPC among HIV-infected adults (clinical cure).

Abbreviations: CLT -Clotrimazole; FLC – Fluconazole; GV – Gentian violet; ITC – Itraconazole; KTC – Ketoconazole; MIC – Miconazole; NYS – Nystatin; PSC - Posaconazole

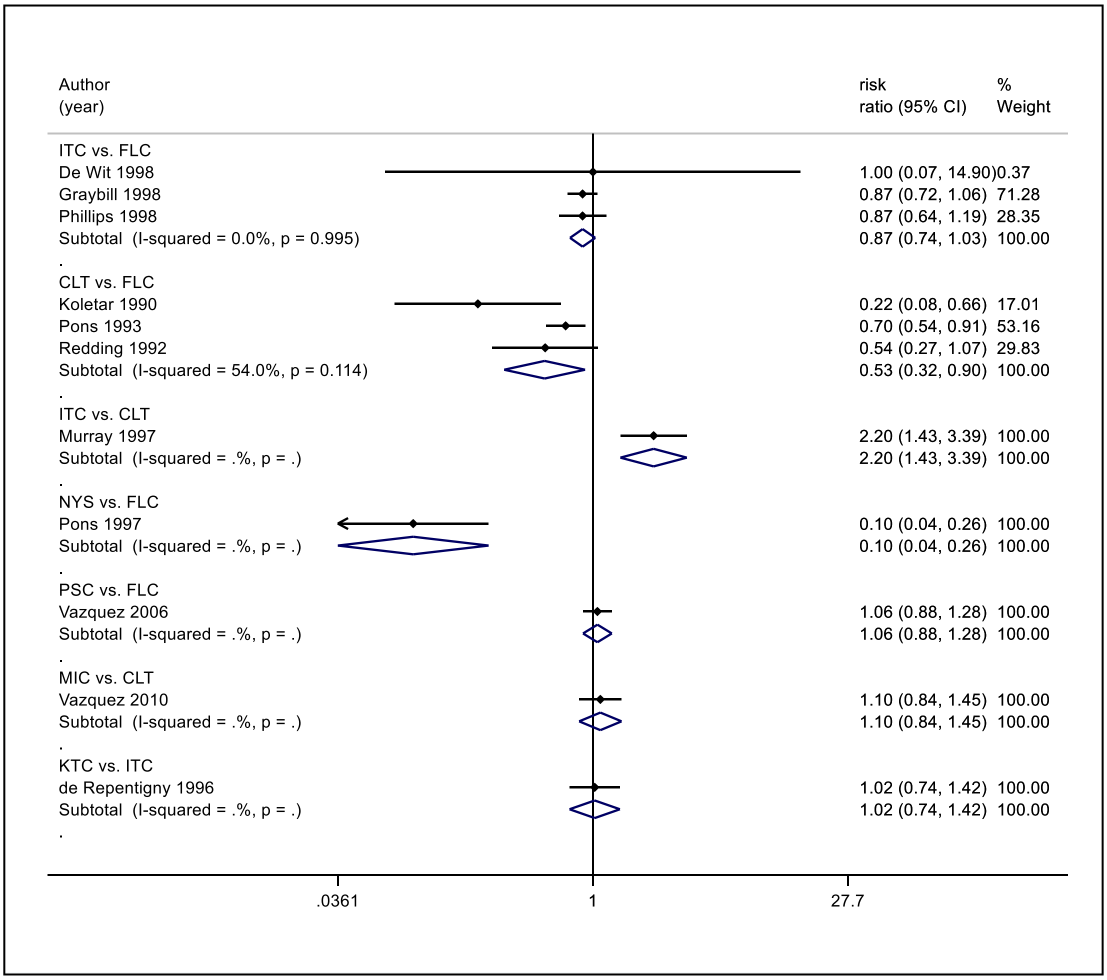

Figure S2. Forest plot of pairwise meta-analysis antifungal agents used for the treatment of OPC among HIV-infected adults (mycological cure).

Abbreviations: CLT -Clotrimazole; FLC – Fluconazole; GV – Gentian violet; ITC – Itraconazole; KTC – Ketoconazole; MIC – Miconazole; NYS – Nystatin; PSC – Posaconazole

CLT	NA	5.33 (0.28,102.26)	NA	NA	NA	NA	1.11 (0.74,1.67)
2.82 (0.26,30.34)	GV	NA	NA	NA	0.87 (0.45,1.69)	NA	NA
1.21 (0.76,1.92)	0.43 (0.04,4.49)	ITC	NA	NA	NA	NA	0.88 (0.70,1.10)
0.83 (0.59,1.17)	0.29 (0.03,3.24)	0.69 (0.38,1.22)	KTC	0.87(0.64,1.19)	NA	NA	NA
0.95 (0.82,1.11)	0.34 (0.03,3.65)	0.79 (0.49,1.28)	1.15 (0.84,1.57)	MIC	NA	NA	1.05 (0.90,1.22)
3.26 (0.33,31.80)	1.15 (0.59,2.25)	2.69 (0.28,25.68)	3.93 (0.39,39.37)	3.41 (0.35,33.50)	NYS	NA	0.33 (0.03,3.10)
1.14 (0.74,1.75)	0.40 (0.04,4.21)	0.94 (0.72,1.24)	1.37 (0.79,2.39)	1.19 (0.76,1.89)	0.35 (0.04,3.31)	PSC	0.94 (0.81,1.09)
1.07 (0.72,1.61)	0.38 (0.04,3.94)	0.89 (0.71,1.12)	1.29 (0.76,2.20)	1.12 (0.73,1.73)	0.33 (0.03,3.10)	0.94 (0.81,1.09)	FLC

Figure S3. Comparative safety of different antifungal agents in treating OPC.

Note: Pairwise (upper right portion) and network (lower left portion) meta-analytic results. Outcomes are expressed as risk ratio (95% confidence intervals). For the pairwise meta-analyses, a relative risk of more than 1 indicates that the treatment specified in the row is safer. For the network meta-analysis, a relative risk of more than 1 shows that the treatment specified in the column is safer. NA- represents there is no direct comparison to show the effect size.

Journal of Fungi

MDPI

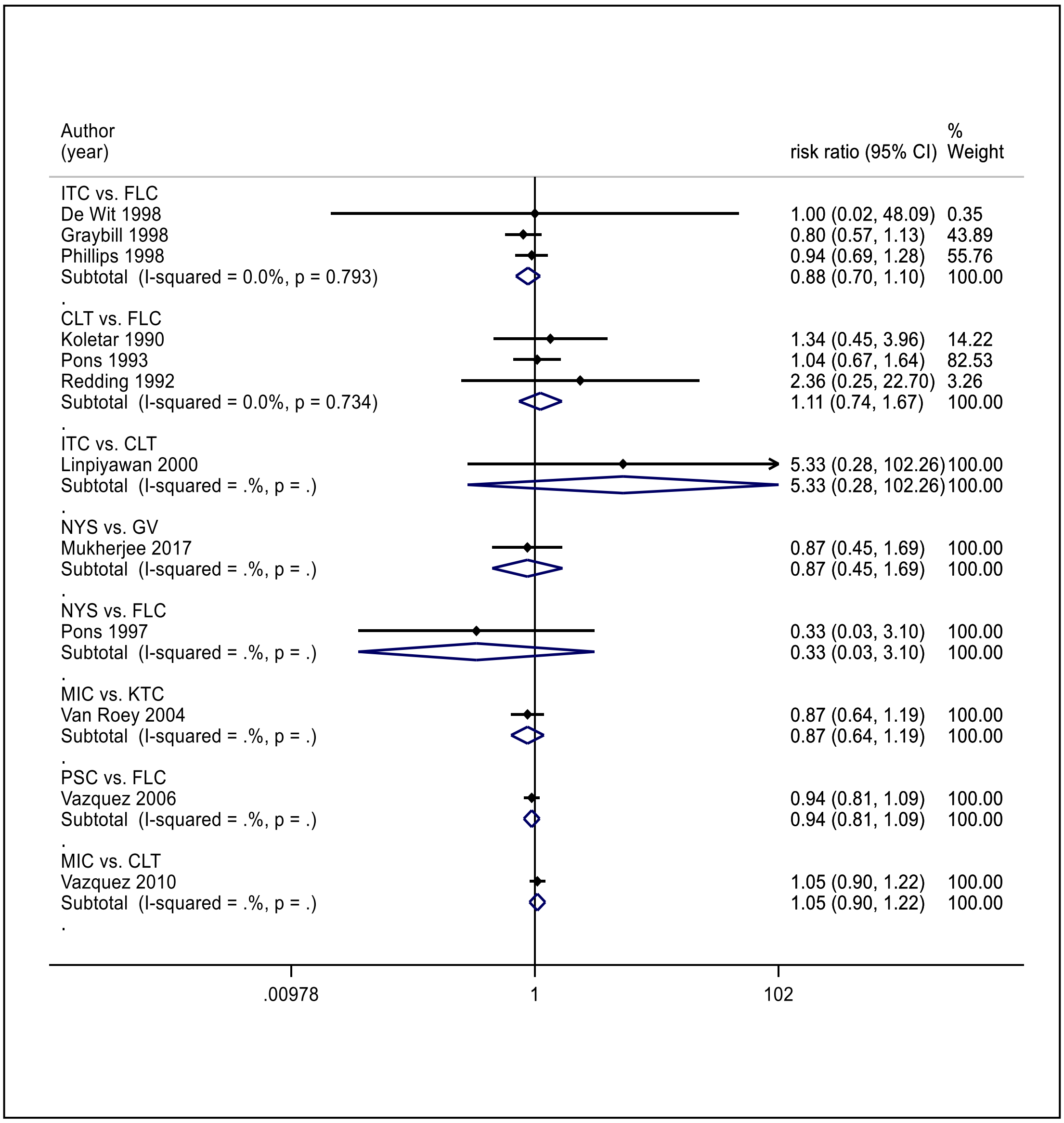

Figure S4. Forest plot of pairwise meta-analysis of the safety of antifungal agents used for the treatment of OPC among HIV-infected adults.

Abbreviations: CLT – Clotrimazole, FLC – Fluconazole, GV – Gentian violet, ITC – Itraconazole, KTC – Ketoconazole, MIC – Miconazole, NYS – Nystatin, PSC – Posaconazole

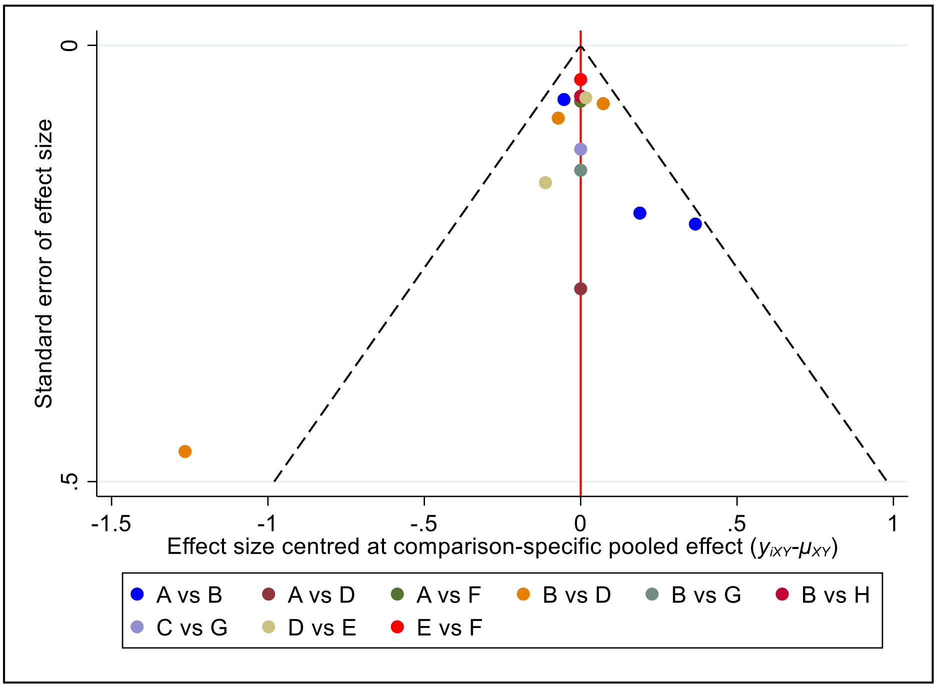

Figure S5. Comparison-adjusted funnel plot of interventions used for the treatment of OPC among HIV-infected adults (clinical cure).

Note: A, Clotrimazole; B, Fluconazole (reference); C, Gentian violet; D, Itraconazole; E, Ketoconazole; F, Miconazole; G, Nystatin; H, Posaconazole.

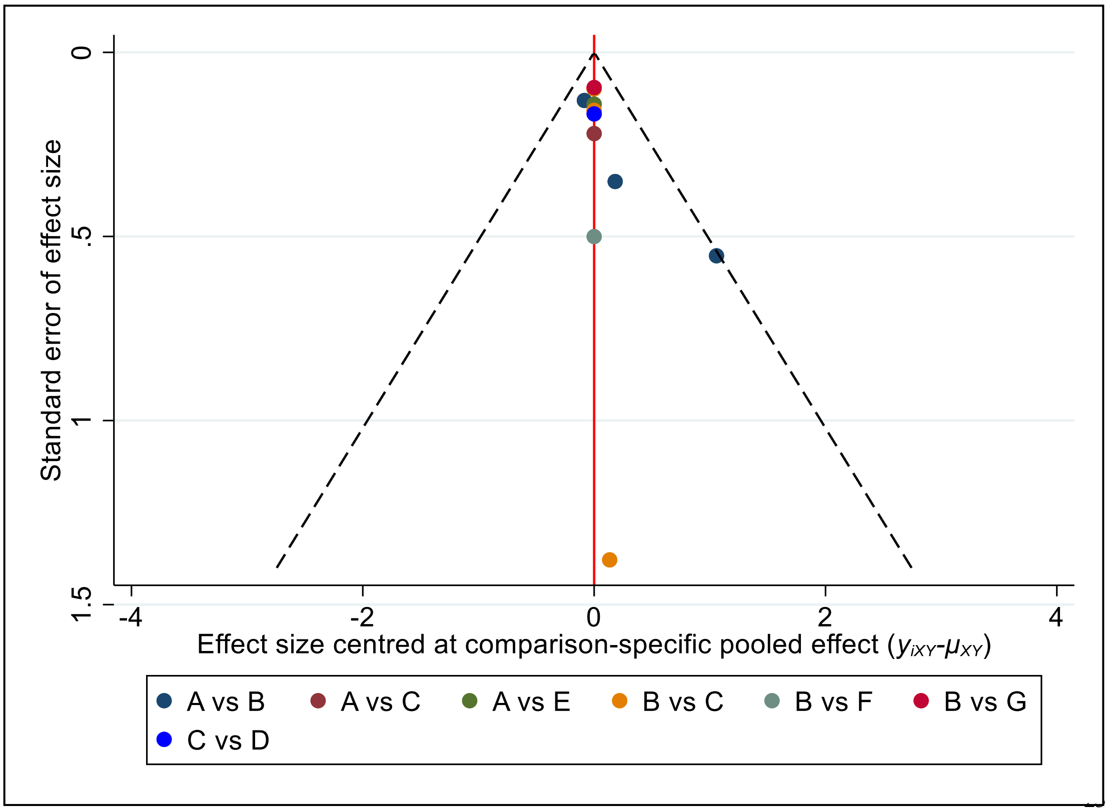

Figure S6. Comparison-adjusted funnel plot of interventions used for treatment of OPC among HIV-infected adults (mycological cure).

Note: A -Clotrimazole, B -Fluconazole (reference), C -Itraconazole, D -Ketoconazole, E -Miconazole, F -Nystatin, G -Posaconazole

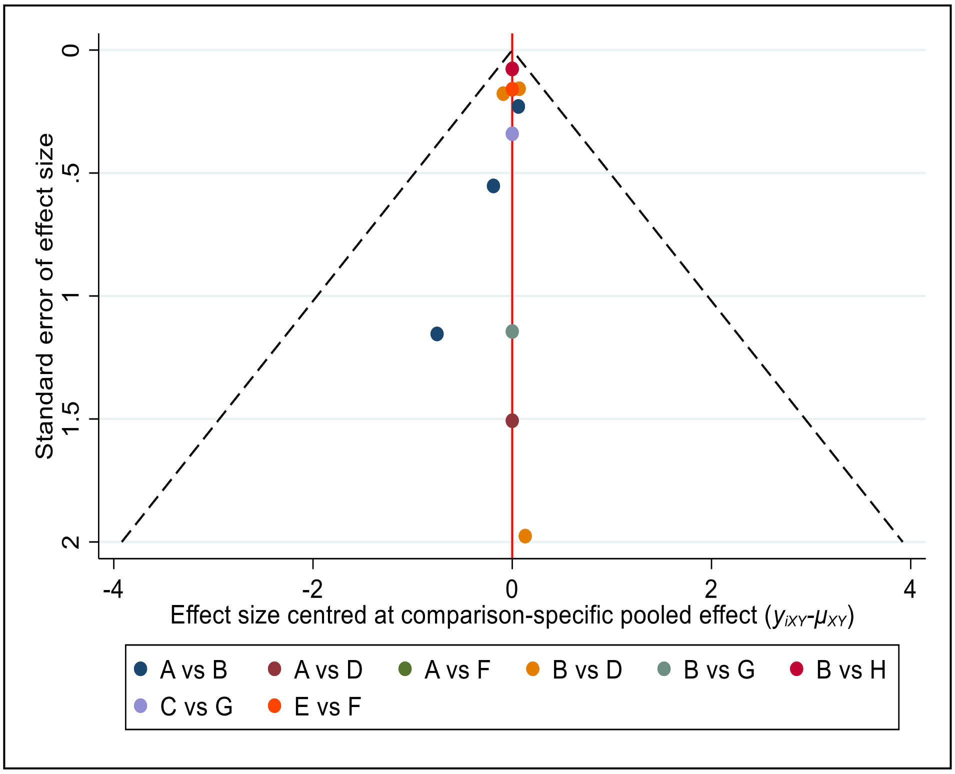

Figure S7. Comparison-adjusted funnel plot of interventions used for the treatment of OPC among HIV-infected adults (safety profile).

Note: A- Clotrimazole, B -Fluconazole (reference), C -Gentian violet, D -Itraconazole, E -Ketoconazole, F -Miconazole, G -Nystatin, H -Posaconazole.
